# Identification of the methionine transporter MetQ in *Streptococcus suis* and its contribution to virulence and biofilm formation

**DOI:** 10.1186/s13567-025-01522-y

**Published:** 2025-05-08

**Authors:** Camila Bosch, Carla García, Luis Saralegui, Lucille van Beek, Marien I. de Jonge, Clara Marín, Jesús Arenas

**Affiliations:** 1 Unit of Microbiology and Immunology, Faculty of Veterinary, University of Zaragoza, Saragossa, Spain; 2 Agrofood Research University Institute of Aragon (IA2), University of Saragossa – CITA, Zaragoza, Spain; 3 Laboratory of Medical Immunology, Radboud Institute for Molecular Life Sciences, Radboud University Medical Center, Nijmegen, The Netherlands; 4 Department of Animal Science, Agrofood Research and Technology Centre of Aragon (CITA), Saragossa, Spain

**Keywords:** *Streptococcus suis*, methionine transporter, ABC transport system, phagocytic activity, biofilm formation

## Abstract

*Streptococcus suis* is a Gram-positive bacterium responsible for various infections in both pigs and humans. This study investigates the role of methionine acquisition in the growth and virulence of *S. suis*. The putative methionine transport system is organised as an operon comprising the *metQ* gene and genes encoding a transposase and an ATPase, forming a typical tripartite ABC transporter. This operon is conserved across multiple streptococcal species, including both animal and human pathogens. We examined whether MetQ functions as a methionine-binding protein and its role in bacterial infection. Using Western blotting and flow cytometry with a specific antiserum, we demonstrated that MetQ is produced in vitro by the *S. suis* reference strain P1/7 under methionine-limited conditions and is located on the bacterial cell surface. Growth assays in chemically defined media revealed that a *metQ* deletion mutant (P1/7∆*metQ*) exhibited impaired growth under methionine-restricted conditions but grew normally in a nutrient-rich medium, suggesting that MetQ primarily transports methionine. Isothermal Titration Calorimetry demonstrated that MetQ binds L-methionine with a dissociation constant (K_D_) of 7.1 µM. In a murine infection model, the *metQ* mutant showed reduced dissemination to internal organs compared to the wild type. Furthermore, the mutant showed decreased intracellular survival in murine macrophages and increased sensitivity to oxidative stress, while exhibited enhanced biofilm formation compared to the wild type. Our findings indicate that MetQ is essential for methionine uptake under methionine-restricted conditions, which is critical for bacterial nutrition, immune evasion, and pathogenicity during infection.

**Supplementary Information:**

The online version contains supplementary material available at 10.1186/s13567-025-01522-y.

## Introduction

*Streptococcus suis* is a Gram-positive bacterium that causes systemic infections in pigs, primarily affecting suckling and nursery pigs. The symptoms include bacteremia, endocarditis, arthritis, pneumonia, and meningitis, which can lead to high mortality rates [1, 2]. Approximately 60–80% of European farms are affected by *S. suis* infections*,* resulting in economic losses estimated at €0.95 per pig due to treatment and prevention costs [3]. This bacterium also poses a zoonotic risk, as it can cause meningitis and septicaemia in humans, particularly among those who handle pigs or consume raw pork [1, 2]. Currently, effective vaccines for *S. suis* infections are unavailable, leading to the overuse of antibiotics and increasing rates of antibiotic resistance worldwide [4], which can result in treatment failure. Overall, *S. suis* represents a significant challenge to both animal and public health.

*S. suis* exhibits substantial genetic and phenotypic diversity. It is classified into 33 serotypes based on capsule antigenicity, with serotypes 2, 9, and 3 being the most prevalent in diseased pigs [5]. Through multi-locus sequence typing [6], more than 3000 sequence types (ST) have been identified globally, with some STs being specific to certain regions [5, 7]. The pathogenicity of *S. suis* involves more than 71 proposed virulence factors [8], although many are specific to certain genetic lineages [9]. This high variability complicates the identification of universally conserved antigens or new antibiotic targets, making it challenging to develop effective vaccines and new drug therapies.

ABC transporters are membrane protein complexes that facilitate the transport of nutrients and other substances across cellular membranes. Several of these transporters are essential for *S. suis* infection [10]. Their typical structure consists of five domains distributed across two to four proteins, forming a functional protein complex. This complex includes two hydrophobic membrane-spanning domains (MSD) that create a substrate translocator channel, two ATP-binding nucleotide-binding domains (NBDs, also called ATP-binding cassettes), and a substrate-binding domain (SBD) that is exposed on the surface of the membrane. The SBD captures and transfers extracellular substrates to the MSDs [11]. Energy from ATP hydrolysis at the NBDs facilitates conformational changes in the MSDs, allowing for unidirectional substrate transport. In many pathogens, ABC transporters play a critical role in nutrient uptake and survival during infection, making them promising targets for antibiotics and vaccines [12].

The gene locus SSU1577 in the *S. suis* strain S735-pCOM1-orf2 was found to be upregulated during experimental infections in pigs [13]. Mutants of this gene in strain 10 exhibited reduced infection capability in competitive co-infection assays and decreased survival in pig blood [13]. Furthermore, deletion of this gene in strain ZY05719 impaired the survival of macrophages [14]. Public genome databases indicate that this gene encodes a predicted SBP, likely part of an ABC transport system involved in methionine uptake. While the importance of this gene in *S. suis* infection is established, its specific role has yet to be demonstrated. This study aims to characterise the biological function of SSU1577 and clarify its role in infection and nutrient uptake.

## Materials and methods

### Bioinformatics

Amino acid sequences encoded by SSU1577 were utilised to predict the cleavage site of the N-terminal signal sequence using the publicly available tool SignalP. We employed the web-based programs AlphaFold and PHYRE2 for predictions of tertiary structures. Protein sequence alignment was performed using MAFFT, while genomic context comparisons were performed with Easyfig.

### Bacterial strains and growth conditions

Table 1 lists all strains used in this study. The *S. suis* strain P1/7, a reference strain isolated from diseased pigs in the UK in 1994, produces a serotype 2 capsule and is classified as ST1 based on MLST. Its genome is publicly available. The strain P1/7Δ*gfp* + is a green fluorescent strain that produces an endogenous green fluorescent protein (GFP) (manuscript in preparation).

**Table 1:** **Strains used in this study**

Strain or plasmid	Relevant characteristics	Source or reference
Strains		
*S. suis*		
P1/7	*S. suis* reference strain, serotype 2, ST1	[15]
P1/7∆*metQ*	P1/7 strain with SSU1577 replaced by *spec*^*R*^	This study
P1/7∆*metQ*/*Q*^+^	P1/7∆*metQ* strain with spec replaced by *metQ* gene and containing *cam*^*R*^	This study
P1/7Δ*gfp* +	P1/7 expressing GFP fused to HlpA with *tet*^*R*^	Laboratory collection
P1/7Δ*gfp* + ∆*metQ*	P1/7Δ*gfp* + with SSU1577 replaced by *spec*^R^	This study
*E. coli*		
BL21 (DE3)	Overexpression strain	Laboratory collection
BL21-pET_MetQ	BL21 (DE3) strain with pET16b_SsMetQ, *Blac*^R^	This study
Plasmid		
pET16b	Plasmid for expression of N-terminally His-tagged recombinant proteins in *E. coli*	Laboratory collection
pET16b-ssMetQ	pET16b derivative encoding recombinant *S. suis* MetQ	This study

*spec*^*R*^ spectinomycin-resistance cassette, *cam*^*R*^ chloramphenicol-resistance cassette, *tet*^*R*^ tetraciclin-resistance cassette, *Blac*^R^ ampicillin resistant.

*S. suis* was grown in Todd-Hewitt Broth (THB, Oxoid Ltd.) or THA (THB supplemented with 15% agar) at 37 °C. When necessary, antibiotics were added to the growth culture to select transformants, including 100 µg mL^−1^ of spectinomycin, 25 µg mL^−1^ of chloramphenicol or 10 µg mL^−1^ of tetracycline. All antibiotics were sourced from Sigma-Aldrich.

For bacterial liquid cultures, colonies grown on THA were transferred to THB at a starting optical density of 600 nm (OD_600_) of 0.05 at 37 °C until reaching an OD_60__0_ of 0.5, indicating exponential growth. The cells were then centrifuged at 4000 rpm for 10 min and resuspended in PBS (7.7 mM Na_2_HPO_4_, 2.3 mM NaH_2_PO_4_, 145.5 mM NaCl, pH 7.3) for further assays.

To evaluate growth under oxidative stress, bacteria from THA colonies were grown in THB supplemented with 2 mM H_2_O_2_ at OD_600_ of 0.02 at 37 °C for 8 h. The number of colony-forming units (CFUs) was assessed by serial dilution of the bacterial suspension in THB, with aliquots propagated on THA plates.

For growth under methionine-restricted conditions, *S. suis* was cultured in a chemically defined medium (CDM) with varying methionine concentrations, following a previously published formula [16]. All components of the CDM were obtained from Sigma-Aldrich. Bacteria from an overnight THB culture were used to inoculate a new THB culture at an OD_600_ of 0.05 in 15 mL tubes. After reaching the logarithmic phase, cells were harvested by centrifugation (4000 rpm during 10 min), washed twice with sterile 0.9% saline solution, and then inoculated into CDM with different methionine concentrations (0 to 100 mg · mL^−1^) in 24-well plates at an initial OD_600_ of 0.05. The plates were covered and incubated at 37 °C for 24 h in a Tecan GENios Pro microplate reader, with OD_600_ monitored automatically. All growth experiments were repeated at least three times in duplicate.

*Escherichia coli* strains were grown in Luria Broth (LB) at 37 °C, supplemented with 100 µg mL^−1^ of ampicillin or 0.1 mM IPTG as needed.

### DNA constructs and preparation of mutants

The primers used in this study are listed in Additional file 1. To prepare knockout mutants, we utilised a recently described technique [17]. DNA fragments upstream and downstream of the SSU1577 locus were amplified from the *S. suis* strain P1/7 chromosomal DNA using high-fidelity DNA polymerase (Thermo Fisher Scientific). Both fragments were then fused to a spectinomycin-resistant cassette through a new PCR reaction using primers that flank the extreme ends.

For genetic complementation, two additional fragments were generated by PCR: one containing SSU1577 and its upstream region and another containing its downstream region of SSU1577. The antibiotic-resistance cassettes were amplified from the chromosomal DNA of antibiotic-resistant strains of *S. suis*, with both fragments being fused to a chloramphenicol-resistant cassette.

We generated fluorescent mutants by amplifying a DNA fragment that codes for a green fluorescent protein from a fluorescent P1/7 strain known as P1/7Δ*gfp* + (unpublished). The PCR reaction consisted of an initial 2-min incubation at 98 °C, followed by 30 cycles of 0.5 min at 98 °C, 0.5 min at 58 °C, and varying elongation times at 72 °C, depending on the specific PCR reaction (Additional File 1). The PCR concluded with a 10-min incubation at 72 °C.

The PCR products were separated on 1% agarose gels and visualised with GelGreen nucleic acid stain (Biotium) in a bioimaging system. After visualisation, the products were purified using a QIAGEN column and subsequently used to transform the *S. suis* strain P1/7 or mutant derivatives (Table 1), as described previously [17, 18].

In brief, a bacterial suspension from an overnight THB culture was used to prepare a 1:40 diluted subculture, which was incubated at 37 °C until it reached an OD_600_ of approximately 0.04. Following this, 1.2 µg of the PCR product was mixed with ComX-Inducing Peptide (XIP) pheromone (GenScript) at a final concentration of 250 µM and incubated at 37 °C for 2 h. The bacteria were then harvested by centrifugation (4000 rpm for 10 min), and the resulting pellet was resuspended in a fresh THB medium. The mixture was plated on THA supplemented with antibiotics and incubated for 12–48 h. Finally, the resulting transformants were analysed by PCR and sequencing to confirm the correct preparation of the mutants.

### Purification of recombinant MetQ and antiserum production

A DNA fragment encoding the mature MetQ of the *S. suis* strain P1/7 was codon-optimised for expression in *E. coli* and cloned into the plasmid pET16b (GenScript). The resulting construct, pET16b-SsMetQ, was used to transform competent *E. coli* BL21(DE3).

To achieve this, the plasmid and *E. coli* cells were mixed and incubated on ice for 15 min, followed by heat shock at 42 °C for 1 min, and then returned to ice for an additional 2 min. Next, 700 µL of LB medium was added, and the mixture was incubated at 37 °C for 30 min. After centrifugation at 4000 rpm for 10 min, the bacteria were plated on THA supplemented with ampicillin. The resulting transformants were used to produce a recombinant MetQ polypeptide (aa 31-283) with an N-terminal His tag, following our earlier reported procedures [19, 20].

A bacterial colony was selected from an overnight culture medium and grown in LB supplemented with 100 µg mL of ampicillin at 37 °C until reaching an OD_600_ of 0.6. Protein production was then induced by adding 0.1 mM IPTG, and the culture was incubated for 2 h. The bacterial cells were harvested by centrifugation (4000 rpm for 10 min) and disrupted by sonication in lysis buffer (50 mM NaH2PO4, 300 mM NaCl, 10 mM imidazole, pH 8.0). The resulting suspension was centrifuged to remove cell debris, and the recombinant MetQ protein was isolated from the supernatant using Ni–NTA agarose chromatography (Quiagen, GmbH) according to the manufacturer’s instructions.

SDS-PAGE validated the correct production and purification of the protein, and its identity was confirmed by western blotting. Protein concentrations were quantified using a 2D quant kit (Sigma-Aldrich).

For antiserum production, 50 µg of purified MetQ were emulsified with TiterMax Gold adjuvant (Sigma-Aldrich) at a 1:1 ratio and injected subcutaneously into mice in two doses, spaced 2 weeks apart. After 2 weeks, the mice were sacrificed using CO_2,_ followed by cervical dislocation, and blood was collected. Following the same immunization schedule, non-immune sera were obtained from animals injected with TiterMax Gold adjuvant mixed with PBS.

### Proteinase K accessibility assays

The accessibility of MetQ and GFP to proteinase K was evaluated following the procedures described in [19, 20]. In brief, *S. suis* strains from logarithmic-phase cultures in CDM supplemented with 5 mg L^−1^ of methionine were incubated with Proteinase K (Fermentas) for 1 h at 37 °C. After incubation, the bacteria were harvested by centrifugation (4000 rpm for 10 min). They were then resuspended in H_2_O, and whole-cell lysates were prepared for western blotting as detailed in the following section.

### Sample preparation, SDS-PAGE, and western blotting

Whole-cell lysates were prepared from bacterial cultures grown to the logarithmic phase, with an estimated OD_600_ of 10 or 1, for *S. suis* or *E. coli*, respectively. The samples were diluted in a fourfold concentrated sample buffer (0.25 M Tris Base, 0.28 M SDS, 40% glycerol, 6 mM bromophenol blue and 3% β-mercaptoethanol), and then boiled for 10 min. SDS-PAGE was performed on 12% polyacrylamide gels using a discontinuous buffer system (0.02 M glycine, 0.025 M base tris, and 0.1% SDS). Electrophoresis was carried out at 200 V for 45 min at room temperature. Following this, the gels were stained with Coomassie Brilliant Blue G250.

In some experiments, proteins were transferred to a nitrocellulose membrane for 60 min at 100 V in a buffer that contained 0.15 M glycine, 0.02 M base Tris, 0.025% SDS and 20% methanol. The blots were stained with Ponceau S (Sigma-Aldrich) to assess transfer efficiency, and then the membrane was washed with PBS to remove the stain. Next, the membrane was blocked with PBS containing 0.1% Tween 20 (PBS-T) and 3% non-fat dried milk for 1 h at room temperature with gentle shaking.

The membrane was incubated overnight with anti-MetQ or anti-His (Invitrogen) antisera, diluted in PBS-T with 0.5% non-fat dried milk. After washing with PBS-T, the membrane was incubated with horseradish peroxidase-conjugated anti-mouse Ig in PBS-T with 0.5% non-fat dried milk. Following another washing step, the blots were developed using the Pierce ECL western blotting substrate and visualised with an iBright 1500 (Invitrogen) imaging system.

### Isothermal titration calorimetry assays

Purified MetQ was measured using far-ultraviolet (UV) spectroscopy (250–700 nm wavelength) on a V-730 Dual Beam UV–visible Absorption Spectrophotometer (Jasco). The maximum absorbance value obtained was used to determine the concentration of MetQ according to the Beer-Lambert law, taking into account its theoretical molar extinction coefficient (E = 31,352.4 M^−1^ cm^−1^).

The protein–ligand interaction was measured using a MicroCal Auto-iTC2000 (Malvern Panalytical). Experiments were conducted in PBS at 25 °C. A 200 µM of L-methionine solution was held in the injection syringe and titrated into a solution containing 38.8 µM purified MetQ within the calorimetric cell. The calorimetric titrations comprised a series of 19 injections, each totalling 2 µL, with a 150 s time interval and an injection speed of 0.5 µL s^−1^. The reference power was maintained at 10 µcal s^−1^ while the stirring speed was set to 750 rpm.

The data from these experiments were analysed using a model that assumed a single ligand-binding site (1:1 stoichiometry), implemented in Origin 7.0 (OriginLab). The binding affinity and enthalpy were estimated through least-squares nonlinear regression data analysis. From these results, the Gibbs free energy and the entropic contribution to binding were calculated using thermodynamic relationships. Given that the model constrains the binding stoichiometry, the parameter “n” reflects a fraction of the protein that is active or competent for binding.

The protein and ligand interaction was measured using a MicroCal Auto-iTC2000 (Malvern Panalytical). Solutions of 38.8 µM and 200 µM of purified MetQ and methionine in PBS were prepared. The reaction was conducted at 25 °C.

### Flow cytometry assays

Flow cytometry assays were conducted using a modified version of a previously described method [21]. Bacteria from exponential growth cultures in CDM containing 5 mg L^−1^ of methionine were harvested and adjusted to a concentration of 10^7^ cells mL^−1^. The bacteria were then fixed in 1% formaldehyde-containing PBS by incubating for 1 h at 37 °C with constant shaking. The bacteria were then harvested by centrifugation (4000 rpm for 10 min) and incubated for 1 h in PBS-T with antiserum at the working dilution at 37 °C with constant shaking.

Following this incubation, the bacteria were again harvested by centrifugation (4000 rpm for 10 min) and incubated with Alexa Fluor 488-conjugated goat anti-mouse IgG (Molecular Probes) diluted in PBS for 1 h at 37 °C with gentle shaking. Fluorescence was measured using a Cytomics FC500 flow cytometer (Beckman Coulter), which was equipped with CXP software, two excitation lasers (an argon-ion laser 488 nm and a solid-state laser 633 nm) and five absorbance filters (FL1-525, FL2-575, FL2-610, FL4-675 and FL5-755). The flow rate was set to 200–300 cells s^−1^, and a minimum of 2 × 10^4^ events was recorded for each measurement.

Bacteria incubated with non-immune mouse serum were used to adjust the instrument for non-specific cell-associated fluorescence. Additionally, unstained bacteria were used to check for overlapping fluorescence. The fluorescence produced by cells incubated with non-immune serum was utilised to adjust the counter so that approximately 75% of the bacteria fell within the 10^1^ fluorescence intensity region. Consequently, the fluorescence intensities reported for immune sera in each experiment were always relative to those obtained using non-immune serum. All experiments were repeated on different days using two replicates per experiment. WinMDI software was used to analyze and present the data.

### Macrophage cultures and phagocytosis assays

The macrophage cell line J774 was cultured in DMEM medium supplemented with 10% non-heat-inactivated fetal calf serum (FCS) and 1% L-glutamine (GlutaMAX). All components of the cell-culture medium were sourced from Gibco. The macrophages were maintained at 37 °C in a humidified atmosphere containing 5% CO₂, using 25-cm^2^ tissue-culture flasks (Nunc) that contained antibiotics (1% penicillin/streptomycin), until they reached ~ 80% confluence.

Two days before the assays, cells from passages 4 to 25 were seeded in 24-well plates. After 24 h of incubation, cells were washed with Dulbecco’s PBS (DPBS; Invitrogen), resuspended in antibiotic-free DMEM medium supplemented with 1% FCS and 1% GlutaMAX, and distributed into 24-well plates. The macrophages were then activated with 1 ng mL^−1^ lipopolysaccharide from *E. coli* (Sigma-Aldrich) and cultivated for 24 h.

To assess macrophage-associated bacteria, bacteria from a logarithmic-phase culture were harvested by centrifugation (4000 rpm, 10 min), suspended in DPBS, and added to the macrophages at a multiplicity of infection (MOI) of 100 and incubated for 2 h. The plates were centrifuged at 1000 rpm for 5 min to enhance the interaction between the cells and the bacteria at the beginning of the incubation. Sequential washes with DPBS removed non-adherent bacteria, and the viable adhering bacteria were counted by determining CFUs after disrupting the cell layer with 1% saponin diluted in DPBS for 20 min, followed by mechanical homogenisation.

To assess intracellular bacteria, 2 h after infection, non-adherent bacteria were removed by washing the macrophages, and the remaining extracellular bacteria were killed using 120 µg mL^−1^ of gentamicin for 1 h. After gentamicin treatment (time 0 and 2 h), the cellular monolayer was treated with 1% saponin for 20 min to release intracellular bacteria, which were then plated on THB for CFU counting.

Cell-associated and extracellular bacteria were evaluated to compare the relative levels of interaction of the strains with the cell monolayers, and their proliferation in the cell culture medium during each experiment, serving as part of our internal controls. Cell-associated bacteria were expressed as a percentage of the initial inoculum. Intracellular bacteria at time 0 were calculated as a percentage of cell-associated bacteria, while intracellular survival at 2 h was expressed as a percentage of intracellular bacteria at time 0.

### Biofilm formation

*S. suis* biofilms were formed according to previously established protocols [22]. In brief, bacteria from exponential-phase cultures were adjusted to an OD_600_ of 1 and placed in 24-well plates (TPP, Techno Plastic Products). These plates were incubated for 4 h, 24 h, and 48 h. After incubation, the supernatant was removed, and the biofilms were washed with deionised water. The biomass was then stained with 0.5% crystal violet and quantified at OD_630_ nm as previously described [19, 21]. Biofilm experiments were conducted on at least four independent days, with three replicates for each experiment. Data were normalised to the wild type, with a value of 1.

For microscopic examination, biofilms were formed on glass, washed, and fixed with 500 µL of PBS containing 2% formaldehyde for 2 h. After washing, the biofilms were visualised using a Leica TSC SP5 inverted microscope equipped with an HCX PLAPO 40x/0.85 objective (Leica Microsystems) as per our experience [19, 23]. Image stacks with 0.4 m z-intervals were acquired, and the structural parameters of the biofilm were analysed using COMSTAT software [24] within the image processing environment ImageJ (v1.48, NHI).

### Animal infections

All animal experiments described here were approved by the Animal Welfare Committee of CITA (TT-SS_2023) and were conducted in accordance with its guidelines and policies. Six-week-old CD-1 mice were obtained from Janvier Labs. The animals were housed in level II facilities at CITA, with feeding and cleaning managed by the staff. Mice were randomly assigned to groups. After a 1-week adaptation period, the animals were briefly exposed to 1% acetic acid. One hour later, the mice were intranasally inoculated with 1 × 10^7^ CFUs of *S. suis* strains. Health, behaviour, and weight were monitored daily to detect clinical signs of streptococcal infection, including depression, swollen eyes, rough hair coat, prostration, and lethargy.

Three days after the onset of infection, the animals were euthanised using CO_2_, followed by cervical dislocation. The nostrils and internal organs (spleen, brain, lungs, heart) were extracted, weighed, and homogenised in a Stomacher 80 (Seward) using sterile bags. The samples were then serially diluted in PBS, and an aliquot from each dilution was propagated on THA and incubated at 37 °C overnight. The number of viable bacteria was determined after 24 h.

### Statistical analysis

Data from at least three independent experiments, each performed in duplicate, were used for statistical comparisons. Data were analysed using an unpaired t-test in GraphPad v6. Statistical significance between groups was considered at *P* < 0.05.

## Results

### Distribution of the *metQ* gene

**Figure 1:**
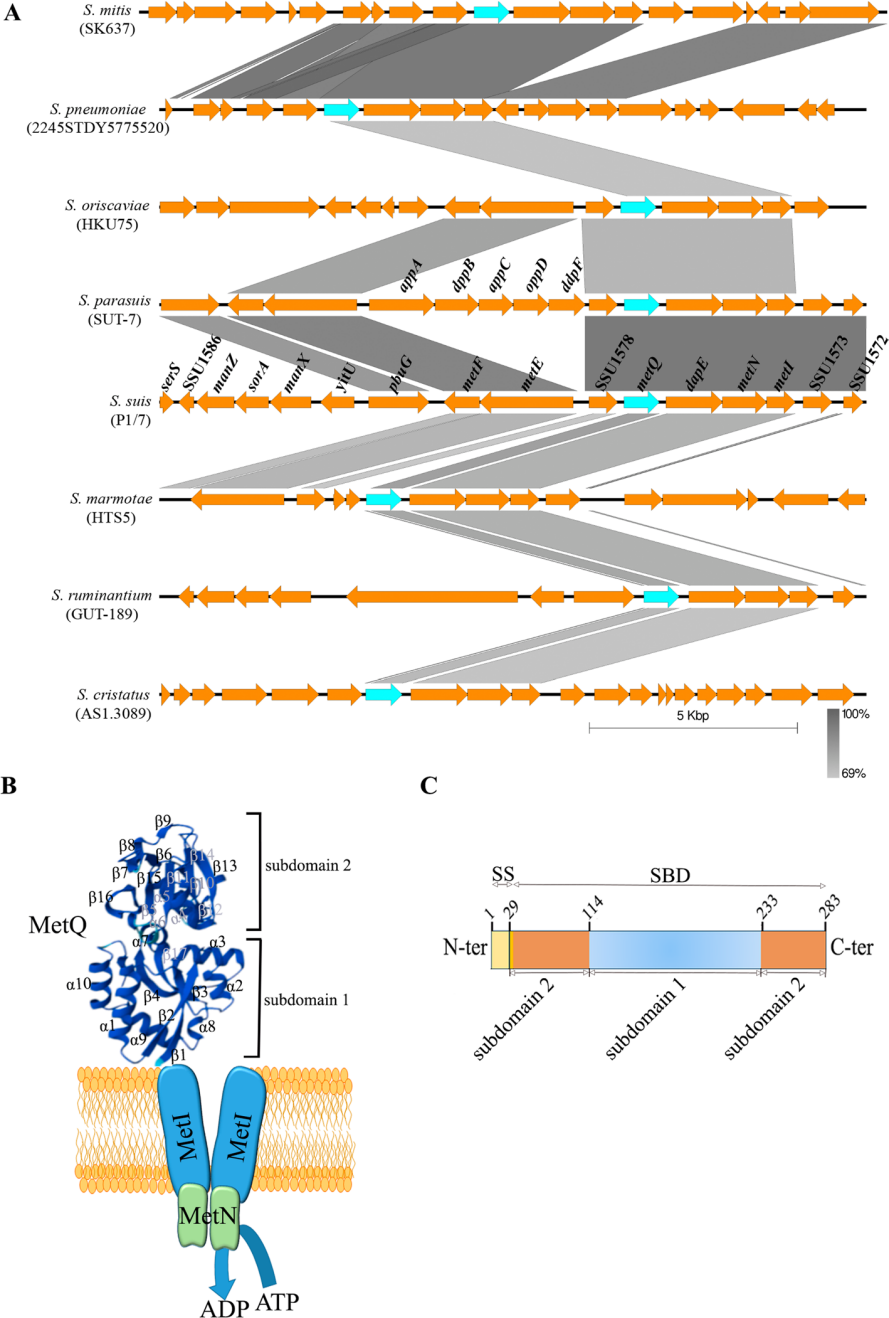
**Schematic representation of MetQ. A** Genetic organization of the methionine transport system in various streptococci. Blue-filled arrows represent putative *metQ* genes, while orange-filled arrows represent other genes assigned in publicly available genomes, along with their proposed systematic names. Gene homology is depicted by shaded regions, with percentage identities indicated within the figure. **B** Predicted tertiary structure of the mature MetQ protein and its location within the methionine ABC transporter complex. The substrate-binding domain (SBD) of MetQ consists of a variable number of α-helices and β-sheets, which are numbered accordingly. **C** Schematic representation of the putative *S. suis* MetQ protein encoded by SSU1577. Predicted organization of the protein domains and their positions within the primary sequence are indicated. The protein includes a signal sequence (SS) for membrane translocation (yellow), followed by an amino acid residue for acylation (lipobox, light orange), and an SBD. The SBD is further divided into subdomain 1 (dark orange) and subdomain 2 (blue).

The locus tag SSU1577 of *S. suis* P1/7 encodes a putative lipoprotein that likely functions as an SBP for methionine (a.k.a. MetQ). In the chromosome of *S. suis* strain P1/7, the *metQ* gene is situated 156 bp upstream of three consecutive genes: *dapE*, *metN*, and *metI.* Among these, *dapE* encodes a putative peptidase from the M20/M25/M40 family of metallo-hydrolases; *metN* encodes an ATPase; and *metI* encodes a permease (Figure 1A). These genes probably form an operon (*metQ/E/N/I*)*.* MetQ, MetN and MetI may together constitute a typical ABC transporter complex (Figure 1B).

DapE acts as a lipoprotein with peptidase activity, converting N-succinyl-L,L-diaminopimelic acid to L,L-diaminopimelic acid and succinate, both of which are part of the biosynthetic pathway for lysine production. The gene at SSU1578, situated 150 bp upstream of *metQ*, encodes Gamma-Glutamyl hydrolase. Furthermore, an operon composed of *metE* and *metF* is located 381 bp upstream of SSU1578, but in the opposite direction. These genes encode 5-methyl-tetrahydropteroyltriglutamate-homocysteine methyltransferase and 5,10-methylenetetrahydofolate reductase, respectively. These enzymes work together to methylate homocysteine, producing methionine by transferring a methyl group from 5-methyltetrahydrofolate.

BLASTn analysis using the *metQ/E/N/I* sequences of *S. suis* as a query shows a sequence homology greater than 96% in public *S. suis* genomes, indicating strong conservation across this species.

The *metQ/E/N/I* operon is found in various streptococcal species. Figure 1A shows its genomic context in representative genomes. In the *S. parasuis* strain SUT-7, an operon consisting of five genes (*appA*, *dppB*, *appC*, *oppD* and *ddpF*) that encode an ABC transport system is located between the gene represented by SSU1578 and the *metE* gene. In many other Streptococcus species that possess the *metQ/E/N/I* operon, however, the *appA* to *ddpF* genes are absent.

The genetic organisation and sequence homology of the genomic context of the *metQ*/*E/N/I* operon vary significantly among species. For example, the *S. ruminantium* strain GUT-189 contains the *metQ/D/N/I* operon but lacks the homologous upstream SSU1578 gene and the *metF*/*metE* operon. In contrast, *S. oriscaviae* possesses both the *metQ/metI* and *metE/F* operons but shares only about 80% homology with the *metE/F* and *metQ/D/N/I* genes. The *S. cristatus* strain AS1.3089 does not have the *metE/F* operon, and its *metQ/D/N/I* operon is disrupted.

The *S. pneumoniae* strain 2245STDY5775520 shares only about 70% sequence homology with the *S. suis met* operon, while the homology in the flanking regions is even lower. However, *S. pneumoniae* shows more than 80% homology with *S. mitis* strain SK637 in the *met* operon and its flanking regions. These analyses indicate substantial genetic rearrangements within the context of the *met* operon across different *Streptococcus* species.

### Structure and variability of MetQ

The MetQ protein from the P1/7 *S. suis* genome consists of 283 amino acid (aa) residues. A BLAST analysis against publicly available *S. suis* genomes indicates over 98% sequence homology, with polymorphisms unevenly distributed throughout the protein sequence. Its N-terminal region contains a signal sequence with a signal peptide (aa 1–29) (Figure 1C and Additional file 2), which is recognised by the Sec machinery for translocation across the membrane. Following this sequence is a cysteine residue (aa 30) that undergoes acylation, anchoring the protein to the membrane (Figure 1C). The mature form of the lipoprotein features an aa-binding domain that spans residues 38–283 (Figure 1C).

Molecular predictions of its tertiary structure using Alphafold suggest that this binding domain consists of two distinct subdomains, connected by a flexible region. The N-terminal subdomain, formed from segments of the N- and C-terminal primary structures, contains five α-helices and five β-sheets arranged in globular configuration (Figures 1B, C). The other subdomain, mainly comprised of the central region of the primary sequence, forms three α-helices and 11 β-sheets of varying lengths, producing a globular but structurally independent unit (Figure 1B). These subdomains are linked by α-helixes, creating a cavity between them.

Comparative structural analysis of the MetQ protein using Phyre2 revealed homologous proteins in other bacterial species, demonstrating 69% structural homology with a methionine transporter from *S. pneumoniae* (PBD: 6jf1) and 59% structural homology with a putative lipoprotein from *S. mutants* (PBD: c4q5tA). Additionally, *S. suis* MetQ shows 38% similarity with a hypothetical membrane protein from *Staphylococcus aureus* (PBD: c1p99A). The crystal structures of MetQ from *Neisseria meningitidis* [25, 26] and *Treponema pallidum* [27] have been determined at resolutions of 2.25 Å and 1.8 Å resolution, respectively, revealing a biobular structure connected by two crossovers with a binding pocket between them. Although these proteins share low sequence similarity with *S. suis* MetQ (Additional file 2), they exhibit a similar predicted structure to *S. suis* MetQ.

Studies on *Treponema* MetQ protein identified three residues involved in substrate binding [27]: Asn116, Glu87, and Arg119. The latter two residues also present in *S. suis* MetQ and in the MetQ proteins of *E. coli*, *N. meningitidis*, and *S. pneumoniae* (Additional file 2). Early research on the substrate‐free and substrate‐bound *N. meningitidis* MetQ highlighted the role of asparagine 238 in ligand binding and affinity [25]. It was demonstrated that both L- and D-methionine interact with residues R156, N213, and N238 located in one domain of MetQ, while residues Y81, F98, H100, and Y103 surround the methionine thioether group. These residues are conserved in the MetQ proteins of *S. suis*, *E. coli*, *Treponema*, and *S. pneumoniae* (Additional file 2). Collectively, these findings support the predicted functional role of the SSU1577 gene product in methionine transport.

### Depletion of *metQ* causes growth reduction under methionine-restricted conditions, and MetQ binds methionine

To determine whether MetQ is necessary for methionine uptake, as suggested by in silico analysis, we tested the growth of the *S. suis* strains P1/7 and its derivative mutant lacking *metQ* known as P1/7Δ*metQ* under methionine-limited conditions. The creation of P1/7Δ*metQ* and the associated results are detailed in Additional file 3.

**Figure 2:**
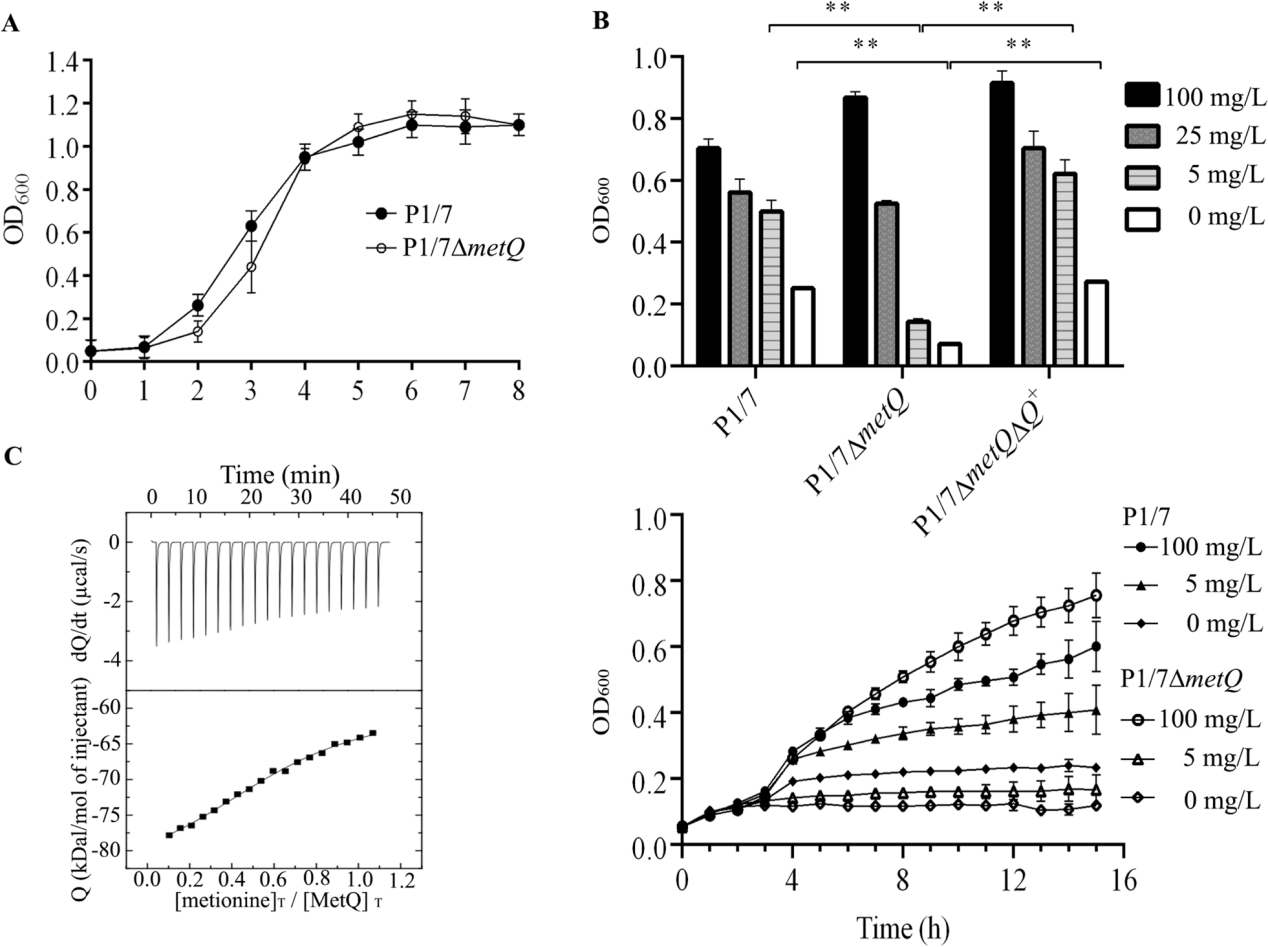
**Impact of MetQ on bacterial growth and methionine binding. A** Growth comparison of *S. suis* P1/7 and P1/7∆*metQ* strains in nutrient-rich THB medium. **B** Growth of P1/7, P1/7∆*metQ* and P1/7∆*metQ∆Q*^+^ after 16 h in a chemically defined medium with varying methionine concentrations. Data represent the average and standard deviation from at least three independent experiments. Statistical analysis was performed using multiple t-tests, indicating significance regarding P1/7 in each condition as **P < 0.01. The lower panel shows a representative growth curve of the most relevant data. **C** Binding isotherms for the interaction of MetQ and L-methionine were measured with isothermal titration calorimetry. The upper panel shows heat changes upon injection of L-methionine and recombinant MetQ protein, expressed as nmol mg^−1^ protein over time, while the lower panel shows integrated heats of injection.

Both strains were initially cultured in an enriched THB medium, where P1/7Δ*metQ* displayed growth comparable to that of P1/7 (Figure 2A). We then evaluated their growth in CDM with varying methionine concentrations (100–5 mg L^−1^) and in methionine-free medium. P1/7 thrived in CDM with 100 mg L^−1^ of methionine, but its growth diminished at 5 mg L^−1^ of methionine or in methionine-free medium (Figure 2B), consistent with previous studies on *S. suis* strain 10 [16].

P1/7∆*metQ* showed similar growth to P1/7 at 100 and 25 mg L-1 methionine concentrations. However, at lower concentrations (5 mg L^−1^) or in the absence of methionine, the mutant exhibited a significant reduction in growth compared to the wild type (Figure 2B). This phenotype was effectively restored upon reintroducing the *metQ* gene into the chromosome (Figure 2B), resulting in a strain called P1/7Δ*metQ*Δ*Q*^+^, which confirms that the observed phenotype is not due to putative adverse effects of the *metQ* mutation.

To further characterise the interaction between MetQ and methionine, we utilised ITC. A recombinant form of mature MetQ, lacking the signal sequence, was generated in *E. coli* (Additional file 2). This recombinant polypeptide was purified via ion exchange chromatography (Additional file 4).

In our experiments, 38.8 µM of the recombinant MetQ was mixed with 200 µM of L- methionine to assess their interaction at 25 °C. The biding reaction yielded a constant of 1.4 × 10^5^ M^−1^ and a binding enthalpy (∆H^0^) corresponding to -104.6 kJ mol^−1^ (Figure 2C), indicating that the reaction is exothermic and spontaneous. Additionally, the affinity curves revealed a dissociation constant of 7.1 µM, demonstrating a high affinity of MetQ for methionine. The changes in entropy (∆S^0^) and Gibbs free energy (∆G^0^) were calculated to be −0.25 and −29.36 kJ mol^−1^, respectively.

### MetQ is produced in *S. suis* and located on the bacterial cell surface

**Figure 3:**
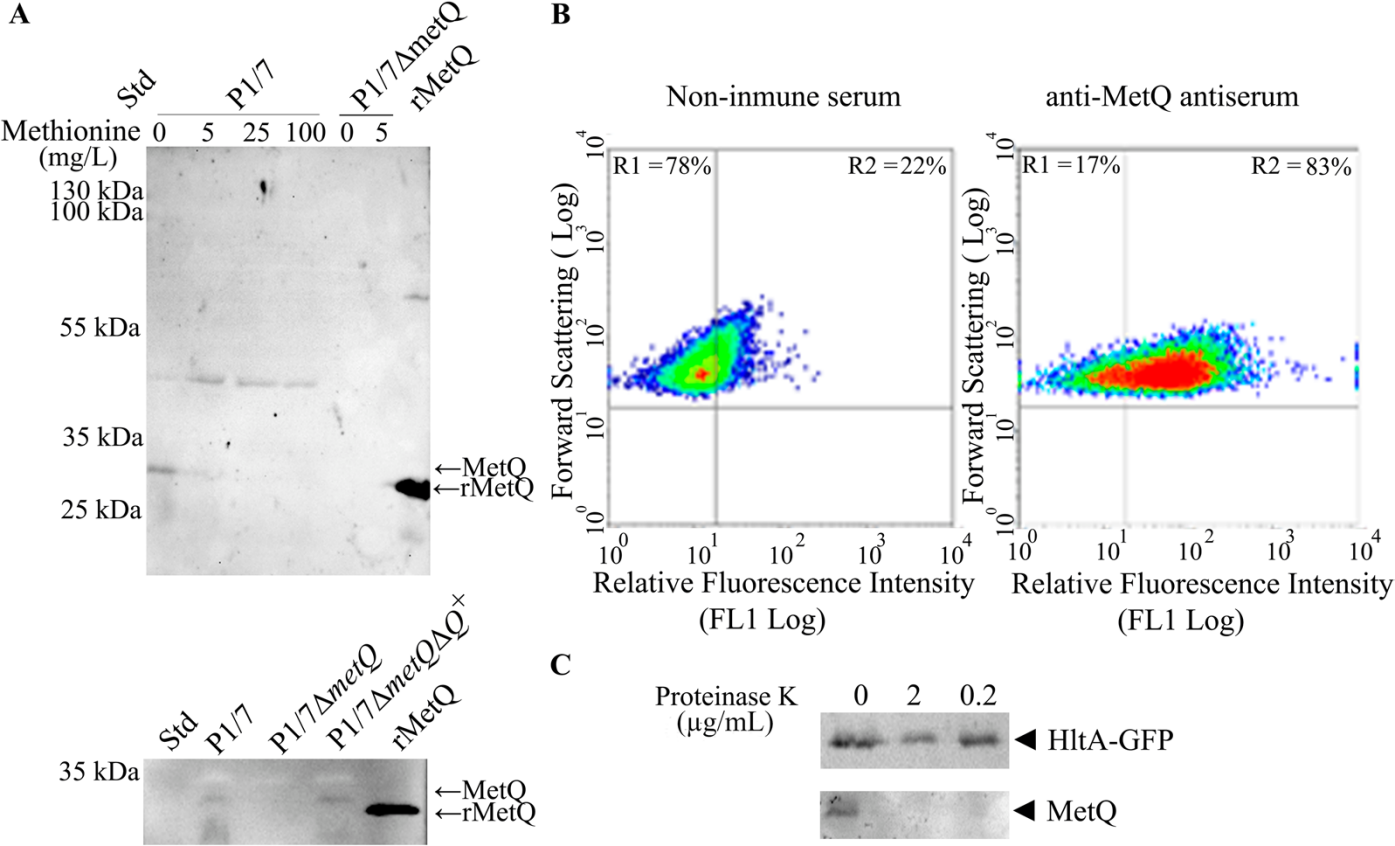
**Production and accessibility of MetQ in *S. suis* P1/7. A** In the upper panel, western blotting analysis of whole-cell lysates from *S. suis* strains P1/7 and P1/7∆*metQ* grown in chemically defined medium (CDM) with varying methionine concentrations, along with recombinant purified MetQ (rMetQ) as a control. In the lower panel, western blotting of whole-cell lysates from *S. suis* strains P1/7, P1/7∆*metQ* and P1/7∆*metQ*∆*Q*^+^ grown in CDM with 5 mg l^−1^ of methionine, also including rMetQ as a reference. The blots were probed with an antiserum against MetQ. **B** Flow cytometry analysis of anti-MetQ antiserum reactivity with formalin-treated cells from *S. suis* P1/7. The percentages of events in selected regions (R1 and R2) are indicated. **C** Proteinase K accessibility assay. Western blotting of whole-cell lysates from intact cells of P1/7Δ*gfp*^+^ treated or untreated with proteinase K probed with anti-MetQ and anti-GFP specific antibodies.

To experimentally confirm the production and localisation of MetQ in the *S. suis* reference strain P1/7, we generated specific antiserum in mice using our recombinant MetQ protein. Western blot assays showed that this antiserum specifically recognised the recombinant MetQ protein (29.3 kDa) (Figure 3A). We detected a band of slightly higher molecular weight in whole-cell lysates of *S. suis* P1/7 grown in CDM with 5 mg L^−1^ of methionine and in methionine-free medium (Figure 3A, upper panel). Without lipid modification, the expected molecular weight of mature MetQ is 28.7 kDa. The presence of this band was confirmed to be specific, as it was absent in P1/7Δ*metQ*, indicating that S. suis P1/7 produced MetQ.

Interestingly, when analysing whole-cell lysates from P1/7 cultures grown in CDM with higher concentrations of methionine (100, 25, and 5 mg L^−1^), we observed a significant reduction in MetQ production compared to the methionine-free medium. This indicates that MetQ production is inversely related to methionine concentration, suggesting it is upregulated under methionine-limited conditions. Furthermore, western blot assays confirmed MetQ production in whole-cell lysates from P1/7Δ*metQ*Δ*Q*^+^ grown in methionine-limiting conditions (Figure 3A, lower panel), supporting the successful restoration of the metQ-dependent phenotype (Figure 2B).

To confirm that MetQ is a surface-exposed lipoprotein, we first conducted flow cytometry on intact bacterial cells of strain P1/7. These cells were incubated with either anti-MetQ antiserum or a non-immune control antiserum, followed by fluorescent labeling with an anti-mouse Ig antibody. The fluorescence analysis of 20 000 bacterial cells showed a significant increase in signal for those incubated with anti-MetQ antiserum, confirming that MetQ is accessible to anti-MetQ antibodies (Figure 3B).

To further investigate MetQ’s accessibility to Proteinase K, we examined P1/7 producing endogenous GFP. Intact cells from growth cultures under methionine-limiting conditions were exposed to various concentrations of Proteinase K. As Proteinase K cannot penetrate the cell membrane, its activity is restricted to proteins on the cell surface. Our results indicated that MetQ was sensitive to Proteinase K concentrations of 0.2 and 2 mg mL^−1^, while the endogenous GFP protein remained resistant (Figure 3C). These findings suggest that MetQ is anchored to the bacterial cell surface, likely due to its lipid modification.

### MetQ is required for *S. suis* virulence in a mouse infection model

Mice infection models are commonly used to assess the attenuation of mutants in putative virulence factors of *S. suis*. We hypothesised that the P1/7*ΔmetQ* strain would show reduced fitness in accessing the bloodstream and disseminating to internal organs compared to the wild-type strain. To evaluate this, we intranasally infected CD1 mice with equally adjusted P1/7 and P1/7Δ*metQ* bacteria suspensions. We monitored classical clinical signs associated with streptococcal disease to confirm the proper progression of the infection.

**Figure 4:**
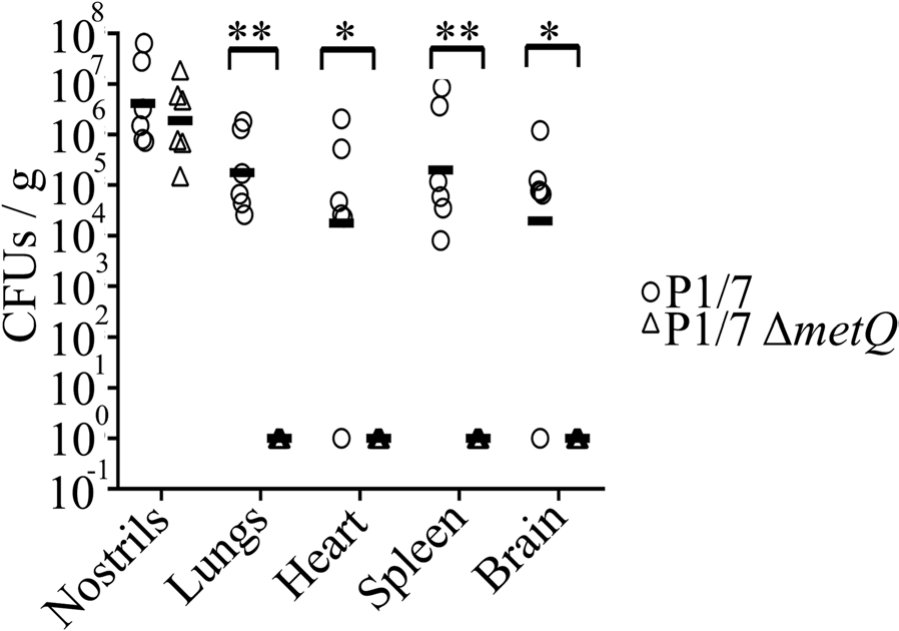
**Effect of *metQ* deletion on *S. suis* virulence in a mouse infection model.** Mice were intranasally infected with bacteria suspensions of *S. suis* strains P1/7 and its *metQ* mutant derivative. Three days post-infection, animals were sacrificed, and samples from the nasal cavity and internal organs were collected. The number of viable bacteria was determined by CFU counting. Statistically significant differences were marked with one (*P* < 0.05) or two asterisks (*P* < 0.01).

After three days post-infection, the mice were sacrificed, and bacterial loads were quantified in the nostrils and internal organs. In the nostrils, we recovered approximately 10^7^ CFUs g^−1^ from P1/7 and P1/7Δ*metQ*-infected mice. However, in internal organs (including the lungs, heart, spleen, and brain), we consistently detected around 10^5^ CFUs g^−1^ of P1/7, while P1/7Δ*metQ* was rarely recovered from any internal organ (Figure 4). These findings demonstrate that MetQ plays a significant role in disseminating *S. suis* during infection.

### MetQ confers protection against the phagocytic activity of macrophages

Phagocytosis is a crucial defence mechanism that helps clear *S. suis* infections. Previous studies have highlighted the role of MetQ in the intracellular survival of both *Neisseria gonorrhoeae* [26] and the *S. suis* strain ZY05719 [14]. This may explain the reduced ability of the *S. suis* P1/7Δ*metQ* variant to infect internal organs in mice (Figure 4).

**Figure 5:**
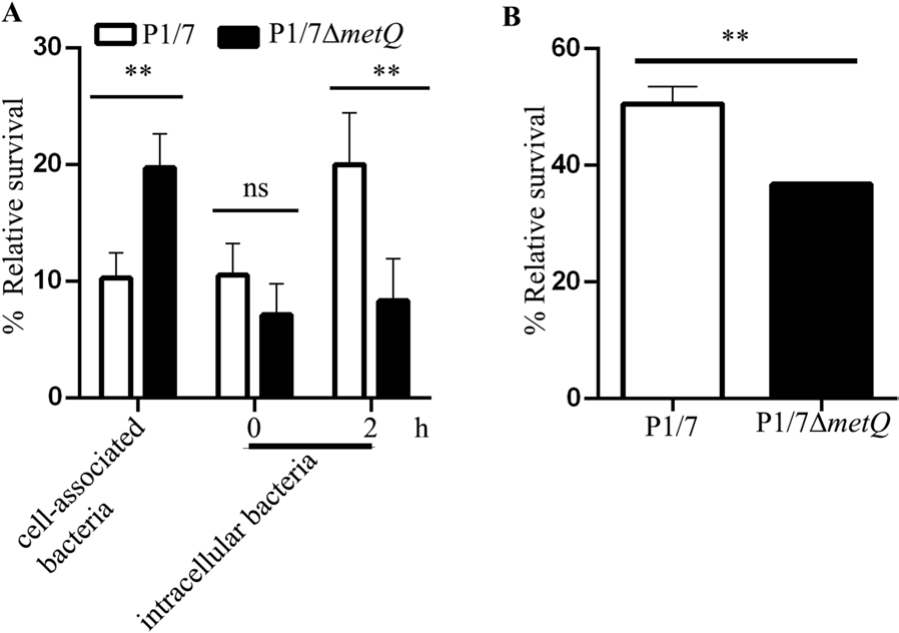
**Effect of *metQ* inactivation on intracellular survival in murine macrophages. A** Macrophages were infected with *S. suis* strains P1/7 and P1/7∆*metQ* for 2 h, washed, and cell-associated bacteria were assessed by CFU counting and refereed with respect to the inoculum. For intracellular bacteria analysis, infected macrophages were treated with gentamicin, and CFU counts for cell-associated bacteria were determined at 0 and 2 h post-treatment. Data shows the percentage of bacteria with respect to cell-associated and intracellular bacteria at time 0, respectively **B** Effect of H_2_O_2_ on bacterial growth. *S. suis* strains P1/7 and P1/7∆*metQ* were grown in THB medium supplemented with 2 mM H_2_O_2_. Data represent the results of three independent experiments and are relativized to growth in THB without H_2_O_2_. Statistically significant differences are marked with two asterisks (*P* < 0.01). ns, no significant differences.

To investigate this further, J774 murine macrophage cell cultures (10^5^ cells) were infected at a MOI 1:100 with suspensions of P1/7 and P1/7Δ*metQ.* The number of cell-associated and intracellular bacteria was analysed after 2 h. Approximately 7.3 × 10^5^ macrophage-associated P1/7 bacteria were detected per well, accounting for about 10% of the inoculum. The P1/7Δ*metQ* strain showed a significantly higher adhesion rate (22%) than P1/7 (10%). However, the percentage of intracellular bacteria at baseline and after 2 h was considerably lower in P1/7Δ*metQ* than in P1/7. Specifically, P1/7Δ*metQ* showed 43.8% and 29.3% fewer intracellular bacteria than P1/7 after 0 and 2 h post-treatment, respectively (Figure 5A).

We hypothesised that the reduced CFU counts of the *metQ* mutant were due to a diminished capacity for intracellular survival, likely stemming from its inability to counteract oxidative stress generated by macrophages. To test this hypothesis, we exposed the bacterial cells to H_2_O_2_, an important reactive oxygen species produced by NADPH oxidase during phagocytosis. H_2_O_2_ can readily penetrate the bacterial cytosol, interacting with ferrous iron to produce reactive hydroxyl radicals, leading to irreversible cellular damage.

To further evaluate this, we examined the survival of P1/7 and P1/7Δ*metQ* in a THB medium supplemented with 2 µM of H_2_O_2._ Notably_,_ P1/7Δ*metQ* exhibited a statistically significant reduction in bacterial survival (34 ± 3.3%) as compared to P1/7 (50.5 ± 3%) (Figure 5B).

### MetQ production contributes to biofilm formation

The high number of cell-associated bacteria observed in the P1/7Δ*metQ* mutant led us to investigate whether MetQ is involved in biofilm formation. This process is crucial for bacterial interactions with surfaces and plays a significant role in host colonisation, antibiotic tolerance and evasion of immune defences [28].

**Figure 6:**
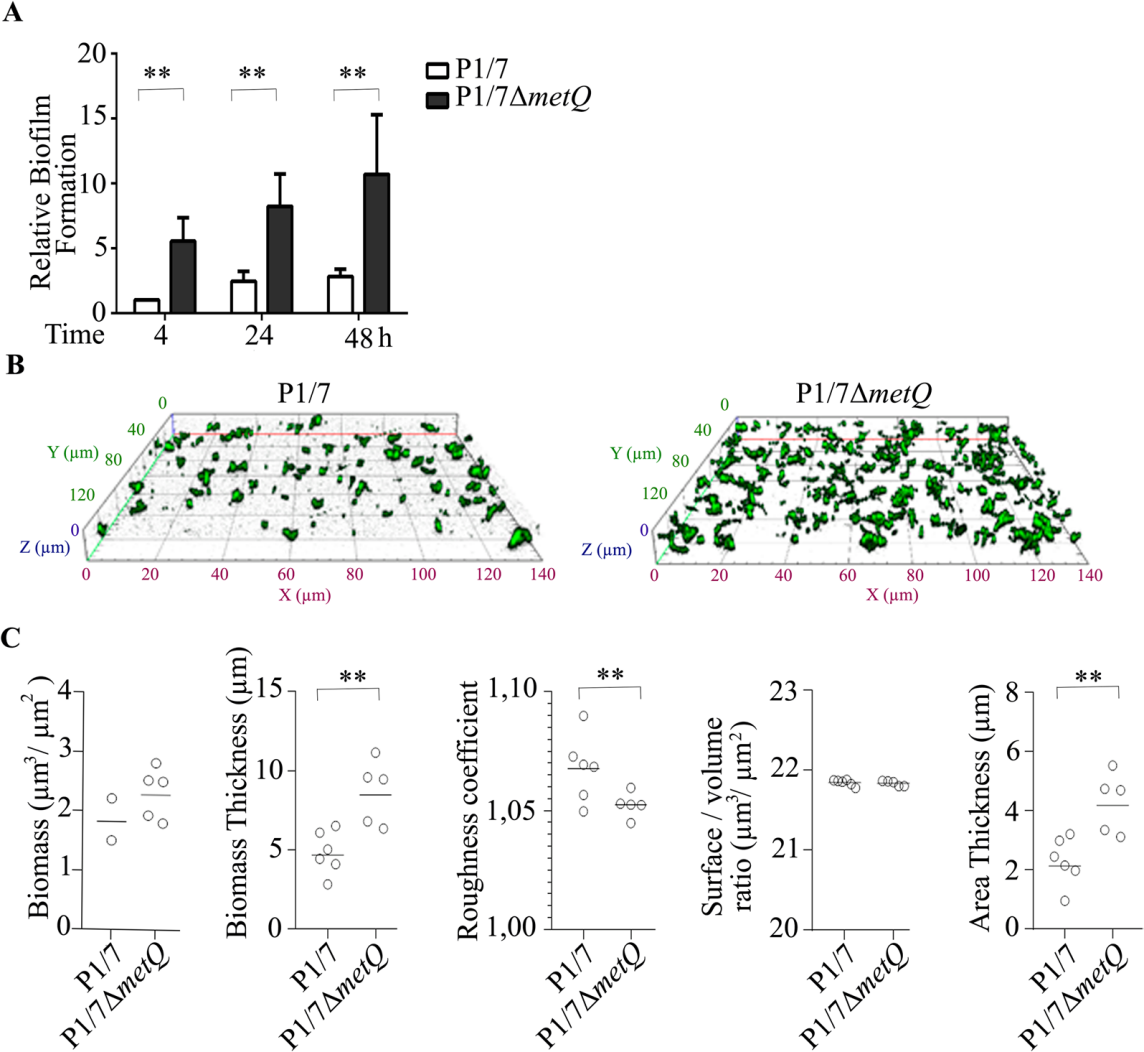
**MetQ mediates *S. suis* biofilm formation. A** Dynamics of biofilm formation of P1/7 and P1/7∆*metQ*. Bacteria were pre-cultured in THB until the end of the logarithmic phase, adjusted to an OD_600_ of 1, distributed in 24-well plates, and incubated for various time periods. Biofilm biomass was quantified using crystal violet, with values relativised to those of P1/7 at 4 h. Data represent the average of at least three independent experiments performed in duplicate, with bars indicating means and error bars showing standard deviation. **B** Spatial organization of 8 h biofilms formed by P1/7 and P1/7 ∆*metQ* visualized through confocal laser scanning microscopy.** C** Biofilm characteristics, including biomass, biomass thickness, roughness coefficient, surface-to-volume ratio, and area thickness for biofilms formed by P1/7 and P1/7∆*metQ*, analysed using COMSTAT. Values represent individual measurements and the average from six image stacks from representative experiments. Statistically significant differences between samples are indicated with two asterisks (*P* < 0.01).

To assess this, we cultured P1/7 and P1/7Δ*metQ* strains on 24-well plates and monitored biofilm formation at 4 h, 24 h, and 48 h using crystal violet staining [22]. Notably, the P1/7Δ*metQ* strain produced four times more biofilm than the P1/7 strain at all time points (Figure 6A), indicating that the absence of MetQ enhances biofilm formation.

We further examined the architecture of the biofilms at 4 h using confocal laser microscopy with genetically engineered GFP-producing strains of both P1/7 and P1/7Δ*metQ*. The biofilms formed by P1/7 exhibited dispersed bacterial clusters with significant gaps between them. In contrast, the biofilms produced by the P1/7Δ*metQ* strain showed large, interacting bacterial clusters that covered a greater surface area than those formed by the wild-type strain (Figure 6B).

Bioinformatic analysis of biofilms using COMSTAT confirmed that P1/7Δ*metQ* biofilms were significantly thicker, had a lower roughness coefficient, and occupied a larger area than wild-type biofilms (Figure 6C). These findings suggest that MetQ plays an essential role in modulating the structure and dynamics of biofilm.

## Discussion

Methionine is essential for protein synthesis and metabolism of phospholipids and nucleic acids [29]. Bacteria acquire methionine through biosynthesis or uptake via permeases and ABC transporters, including MetQ/L/N. This system is widely conserved across Gram-positive and Gram-negative bacteria. It has been identified as the primary methionine transporter in several species, such as *Bacillus subtilis* [30], *S. pneumoniae* [31], or *S. aureus* [32].

We identified the gene *metQ* (SSU1577) in *S. suis*, which is part of an operon that includes *metL/N*. This composition differs from the typical methionine ABC operon found in many bacteria, which generally consists of *metL/N/Q*. In *S. suis*, *metQ* is located upstream in the operon, followed by *dapE* and *metL/N* (Figure 1A). This positioning suggests that *metQ* may have experienced genetic rearrangements.

Variations in the canonical *met* operon are not exclusive to *Streptococcus*. For example, members of the Lactococcus genus, such as the *Lactococcus lactis* strain LAC460, exhibit multiple *metQ*-like genes upstream of the putative *metL/N* genes. Some *metQ* homologs share over 80% sequence identity, indicating possible gene duplication events.

Furthermore, the presence of the *dapE* gene, which encodes an enzyme involved in the lysine biosynthetic pathway, within the *met* operon of *Streptococcus* is noteworthy. Early studies have shown that *dapE* has aspartyl peptidase activity [33], suggesting it may play a role in releasing methionine residues from degraded polypeptides for uptake by the Methionine ABC transport system.

Bioinformatic analysis revealed that *S. suis* MetQ possesses conserved substrate-binding residues and shows structural similarities to experimentally validated methionine-binding proteins despite having low sequence similarity (Figure 1B, C and Additional file 1). Deletion of the *metQ* gene impaired growth under conditions of methionine restriction (Figure 3B), confirming its role in methionine uptake. Complementation with the wild-type gene restores the normal phenotype, contrasting with previous studies in *S. pneumoniae* [31], where genetic complementation of the homologous *metQ* mutant was unsuccessful. Additionally, earlier research on the *S. suis* strain ZY05719 *metQ* mutant failed to demonstrate genetic complementation [14]. Our ITC assays indicated methionine binding, albeit with a lower affinity than *S. pneumoniae* MetQ [31]. These differences may arise from variations in aa sequences (Additional file 1), potentially altering its interaction with methionine. Finally, we observed that MetQ production was upregulated under conditions of methionine restriction (Figure 3A), further supporting its role in methionine uptake. Collectively, our findings confirm that the SSU1577 locus of *S.* *suis* strain P1/7 encodes MetQ.

Methionine metabolism plays a significant role in the virulence of various pathogens, including *Brucella melitensis* [34], *Haemophilus parasuis* [35], *Streptococcus* B [36] and *Salmonella Typhimurium* [37]. Our infection studies in mice indicate that *S. suis* MetQ is also essential for full virulence. These findings align with our earlier results from pig infection models [13], where a *metQ* mutant of the *S. suis* strain 10 (serotype 2) demonstrated reduced virulence when competing with the wild-type strain.

Several factors may explain the role of MetQ in the virulence of *S. suis*. First, the methionine concentration in mammalian physiological fluids is low, ranging from 1 to 7 µg mL^−1^. Consistent with this finding, we observed a reduced growth rate of our *S. suis* P1/7Δ*metQ* mutant at low methionine concentrations in CDM (Figure 2B). Moreover, previous studies have shown that a *metQ* mutant in *S. suis* strain 10 exhibited impaired growth in pig blood [13], suggesting that the inability to acquire methionine from the bloodstream likely reduces *S. suis* growth during bacteremia, limiting its capacity to cause infection.

Second, restricted growth due to limited methionine availability may also impact the expression of virulence factors. For instance, faster bacterial growth rates have been associated with increased capsule expression in *Streptococcus* Group B [38]. The capsule is a well-known virulence factor in *S. suis*, as it inhibits phagocytosis [39]. Supporting this hypothesis, our macrophage assays revealed that the *S. suis* P1/7Δ*metQ* displayed higher adhesion to macrophages than the wild-type strain (Figure 5A). However, P1/7Δ*metQ* showed reduced intracellular survival following bacterial uptake. Similar observations have been reported with the *S. suis* strain ZY05719 [14] and the Gram-negative bacterium *Neisseria gonorrhoeae* [40], further confirming our results. Quantitative proteomics of macrophages infected with *S. suis* demonstrated that MetQ influences the macrophage response to phagocytosed *S. suis* [14] by regulating proteins involved in actin cytoskeleton dynamics, focal adhesion, and chemokine signalling pathways.

Additionally, methionine acts as an endogenous antioxidant due to its reversible reduction–oxidation capacity. Therefore, the limited methionine levels in the P1/7Δ*metQ* mutant may compromise the bacteria’s resistance to oxidative stress during phagocytosis. Our results indicate that the *S. suis* P1/7Δ*metQ* mutant exhibited lower survival rates when exposed to H_2_O_2_ exposure than the parent strain (Figure 5B). Overall, our findings support the hypothesis that MetQ contributes to bacterial virulence by facilitating optimal nutrition within the host and enhancing resistance to phagocytosis-induced oxidative stress.

We demonstrated for the first time that MetQ in *S. suis* plays a crucial role in regulating biofilm formation. Biofilm formation is vital for host colonisation and is associated with infections of various internal organs, including endocarditis. Additionally, biofilms contribute to antimicrobial tolerance [28], enhancing bacterial persistence within the host. Our findings indicate that MetQ influences the architecture of *S. suis* biofilm, aiding in the organisation of microcolonies (Figure 6).

To the best of our knowledge, the role of MetQ in biofilm formation has not been previously reported in other bacteria. For example, studies on *Neisseria gonorrhoeae* suggest that MetQ may function as an adhesin [40]. This finding contrasts with our results, as we observed an increase in biofilm formation in the absence of *metQ*, which is the opposite of what would be expected based on the findings in Neisseria. However, our experiments were conducted on abiotic surfaces, lacking eukaryotic cells, where the putative receptor for MetQ is missing.

In *S. pneumoniae*, methionine limitation triggered significant changes in the transcriptome [41, 42]. The differentially expressed genes included many involved in methionine biosynthesis and others with no apparent connection to methionine metabolism or transport. One of the most enriched molecules under methionine restriction was cAMP [41], a second messenger primarily involved in carbon catabolite repression in bacteria. High levels of cAMP have been shown to promote biofilm formation in several pathogens, including *E. coli*, *Klebsiella pneumoniae*, and *Pseudomonas aeruginosa* [43].

For instance, in *K. pneumoniae*, cAMP enhances the synthesis of type 3 fimbriae, while in *P. aeruginosa*, it stimulates the production of Type IV pili. Both of these substances are major adhesins involved in interbacterial and bacteria-substrate interactions during biofilm formation. Interestingly, Type IV pili are also produced by Streptococcal species, and their role in biofilm formation has been demonstrated in *Streptococcus sanguinis* [44].

Thus, we speculate that MetQ's role in biofilm formation may be linked to reduced intracellular methionine levels, which could influence the expression of a wide range of factors involved in biofilm formation. Further investigation is needed to identify these factors and understand their regulation in *S. suis*. Our study establishes that MetQ is key in methionine uptake, virulence, oxidative stress resistance, and biofilm formation in *S. suis*.

## Supplementary Information

**Additional file 1. Primers used in this study.**

**Additional file 2. Alignment of MetQ proteins across various bacterial species.** The alignment includes MetQ from *S. suis *strain P1/7 (SSU1577), *S. pneumoniae* strain 284 (ACGQOV_002047), *Neisseria meningitidis* strain BZ133 (GNA1946), *Escherichia coli* strain NCTC9081 (NCTC9081_02188), and *Treponema pallidum* strain TPANIC_082. Residues highlighted in red indicate amino acids involved in substrate binding as identified in *T. pallidum*. A residue highlighted in green indicates an essential substrate binding site identified in *E. coli* and *N. meningitidis*. Residues involved in protein substrate interaction, as identified in *N. meningitidis*, are marked in red. An asterisk indicates a residue conserved across all strains, a single dot marks residues shared by at least two strains, and two dots mark residues shared by three or more strains. Green arrows indicate the start and end points of the sequence of the recombinant MetQ, which is fused in its N-terminal extreme to MCGGHHHHHHHH, as encoded by the plasmid.

**Additional file 3. Confirmation of the metQ knock-out mutant**. (A) Schematic representation of the *metQ* gene context in *S. suis* strain P1/7 and P1/7Δ*metQ* mutant, indicating the position of the primers used for PCR validation. Agarose gels showing the PCR products amplified using primers (B) *metQ*_L-Fw and metQ_R-Rev and (C) metQ_Fw and metQ_Rev primers in both the wildtype and Δ*metQ *mutant. Primer sequences and predicted amplicon sizes are specified in Additional file 1.

**Additional file 4. Production of recombinant MetQ.** (A) SDS-PAGE assays showing whole cell lysates of BL21, BL21 carrying p16b-MetQ (BL21-pMetQ) grown in the presence or absence of IPTG and purified recombinant MetQ (rMetQ). (B) Western blot analysis of whole cell lysates from BL21, BL21-pMetQ grown in the presence or absence of IPTG and rMetQ using antiserum directed against a Histidine - tag. The position of rMetQ is indicated. Std (Standard Molecular weight).

## Data Availability

The data generated in this work are available in this paper or in the additional files.
