# Supplementary material for: Identification of the methionine transporter MetQ in Streptococcus suis and its contribution to virulence and biofilm formation
Source: Vet Res. 2025 May 8;56:99. doi: 10.1186/s13567-025-01522-y (PMC12063423; doi:10.1186/s13567-025-01522-y)
Supplement: Supplementary file 1 — Additional file 1. Primers used in this study. [file 13567_2025_1522_MOESM1_ESM.pdf]

Additional file 1. Primers used in this study.

Goal	Name	Sequence (5' to 3')	Amplicon size (bp)	Elongation time in PCR (min)
metQ gene detection	metQ_Fw	TACCAAACTGGCTCATCCAT	705	0.5
	metQ_Rev	GCCGTTGGGATAAAGTTCAA		
Generation of *metQ* mutant or complemented strain: downstream flanking segment amplification	metQ_L-Fw	GCGCGCGCGCAGATCTGAACCTGATCGTAAATGCCT	932	0.5
	metQ_L-Rev	**AGTCAGTCGATCACAACTAT**GCGCAAATAGGTTGAGGACTGGGC		
Generation of *metQ* mutant: upstream flanking segment amplification	metQ_R-Fw	**AGTCTACGTACATAGTCAGT**TAAAGCAGCCGTTGCCAAAC	988	0.5
	metQ_R-Rev	GCGCGCGCGCGCGCGATATCATATTTTTTATCAAGCCATA		
Generation of *metQ* complemented strain: upstream flanking segment amplification	metQ_R_Fw_c	**AGTCTACGTACATAGTCAGT**TTACCAAACTGGCTC	1799	1
	metQ_R-Rev	GCGCGCGCGCGCGCGATATCATATTTTTTATCAAGCCATA		
Spectinomycin-resistance cassette amplification	SPECT_Fw	**ATAGTTGTGATCGACTGACT**GCAGGTCGATTTTCGTTCGTG	1199	1
	SPECT_Rev	**ACTGACTATGTACGTAGACT**ATGCAAGGGTTTATTGTTTTCTAAAA		
Chloramphenicol-resistance cassette amplification	CAM-Fw	**ATAGTTGTGATCGACTGACT**AAGGGATCCGGCACCTATCT	1196	1
	CAM-Rev	**ACTGACTATGTACGTAGACT**TGCCCGGGGATCCTCCGATA		
Amplification of hlpA-GFP-Tet^R construct	GFP-Fw	TCCACTAATAGGGAACAGCT	4799	3
	GFP-Rev	ATGAACAGAGACAAGCTGTC		
Confirmation of (1) wild type, (2) *metQ* mutant or (3) complemented strains.	metQ_L-Fw	GCGCGCGCGCAGATCTGAACCTGATCGTAAATGCCT	(1) 2695<br>(2) 3079<br>(3) 3887	2.5
	metQ_R-Rev	GCGCGCGCGCGCGCGATATCATATTTTTTATCAAGCCATA		
