# Supplementary material for: Identification of the methionine transporter MetQ in Streptococcus suis and its contribution to virulence and biofilm formation
Source: Vet Res. 2025 May 8;56:99. doi: 10.1186/s13567-025-01522-y (PMC12063423; doi:10.1186/s13567-025-01522-y)
Supplement: Supplementary file 2 — Additional file 2. Alignment of MetQ proteins across various bacterial species. The alignment includes MetQ from S. suis strain P1/7 (SSU1577), S. pneumoniae strain 284 (ACGQOV_002047), Neisseria meningitidis strain BZ133 (GNA1946), Escherichia coli strain NCTC9081 (NCTC9081_02188), and Treponema pallidum strain TPANIC_082. Residues highlighted in red indicate amino acids involved in substrate binding as identified in T. pallidum. A residue highlighted in green indicates an essential substrate binding site identified in E. coli and N. meningitidis. Residues involved in protein substrate interaction, as identified in N. meningitidis, are marked in red. An asterisk indicates a residue conserved across all strains, a single dot marks residues shared by at least two strains, and two dots mark residues shared by three or more strains. Green arrows indicate the start and end points of the sequence of the recombinant MetQ, which is fused in its N-terminal extreme to MCGGHHHHHHHH, as encoded by the plasmid. [file 13567_2025_1522_MOESM2_ESM.pdf]

```
                     signal peptide
S. suis          MKLK-KLFSLATAALSVGV-LAACGNSSSSSDS-----------SATTVRVGVMSLSDSE
S. pneumoniae    MKIK-KWLGLAALATVAGLALAACGNSEKKAD------------NATTIKIATVNRSGSE
N. meningitidis  MKTFFKTLSAAAL----ALILAACGGQKDSAPAASASAAADNGAEKKEIVFGTT-VGDFG
E. coli          MAFKFKTFAAVGALIG-SLALVGCGQDEKDPN---------------HIKVGVI--VGAE
T. pallidum      MKGKTVSAALVGKLIALSVGVVACTQVKDE-----------------TVGVGVL--SEPH
                 *       . .      .: :..*   ...                  : ...

S. suis          QARWDKVQEIL--GDEVKLEFTQFTDYSQPNKAVAENEVDINAFQHYNFLNNWNQENGED
S. pneumoniae    EKRWDKIQELVK-KDGITLEFTEFTDYSQPNKATADGEVDLNAFQHYNFLNNWNKENGKD
N. meningitidis  DMVKEHIQPELE-KKGYTVKLVEFTDYVRPNLALAEGELDINVFQHKPYLDDFKKEHNLD
E. coli          QQVAEVAQKVAKDKYGLDVELVTFNDYVLPNEALSKGDIDANAFQHKPYLDQQLKDRGYK
T. pallidum      ARLLEIAKEEVK-KQHIELRIVEFTNYVALNEAVMRGDILMNFFQHVPHMQQFNQEHNGD
                     :  :          :.:. *.:*   * *   .::  * ***  .:::  ::.. .

S. suis          LVAIADTYIAPIRLYSGTADGKNKYTKVEEIPDGAEIAVPNDPTNESRALYLLQAAGLIK
S. pneumoniae    LVAIGDTYISPIRLYSGLNGSANKYTKVEDIPANGEIAVPNDATNESRALYLLQSAGLIK
N. meningitidis  ITEVFQVPTAPLGLYPG------KLKSLEEVKDGSTVSAPNDPSNFARVLVMLDELGWIK
E. coli          LVAVGNTFVYPIAGYSK------KIKSLDELQDGSQVAVPNDPTNLGRSLLLLQKVGLIK
T. pallidum      LVSVGNVHVEPLALYSR------TYRHVSDFPAGAVIAIPNDSSNEARALRLLEAAGFIR
                 :. : :.   *:  *.       .   :.:.  .. :: ***.:* .* * :*:  * *:

S. suis          VGVSGT-ELATIADITENKKNLKITELDASQTASSLS--SVDAAVVNNTFVLEAGLDYK-
S. pneumoniae    LDVSGT-ALATVANIKENPKNLKITELDASQTARSLS--SVDAAVVNNTFVTEAKLDYK-
N. meningitidis  LKDGINPLTASKADIAENLKNIKIVELEAAQLPRSRA--DVDFAVVNGNYAISSGMKLT-
E. coli          LKDGVG-LLPTVLDVVENPKNLKIVELEAPQLPRSLDDAQIALAVINTTYASQIGLTPAK
T. pallidum      MRAGSG-LFATVEDVQQNVRNVVLQEVESALLPRVFD--QVDGAVINGNYAIMAGLSARR
                 :  .     .:  :: :* :*: : *:::.  .      .:  **:* .:.    :

S. suis          NALYKEQKDENSKQWYNLIAARSDWEKSEQAAAIKKIIEAYHTDEVKKVIEETSDGMDEP
S. pneumoniae    KALFKEQADENSKQWYNIIVAKKDWETSPKADAIKKVIAAYHTDDVKKVIEESSDGLDQP
N. meningitidis  EALFQE----PSFAYVNWSAVKTA---DKDSQWLKDVTEAYNSDAFKAYAHKRFEGYKSP
E. coli          DGIFVE---DKESPYVNLIVTRED---NKDAENVKKFVQAYQSDEVYEAANKVFNGGAVK
T. pallidum      DGLAVE---PDASAYANVLVVKRG---NEADARVQAVLRALCGGRVRTYLKERYKGGEVA
                 ..:  *        : *  ..:     .     :: .  *   . .    .:  .*

S. suis          V-W------
S. pneumoniae    V-W------
N. meningitidis  AAWNEGAAK
E. coli          G-W------
T. pallidum      PAL------
```

Additional file 2. Alignment of MetQ proteins across various bacterial species. The alignment includes MetQ from *S. suis* strain P1/7 (SSU1577), *S. pneumoniae* strain 284 (ACGQOV_002047), *Neisseria meningitidis* strain BZ133 (GNA1946), *Escherichia coli* strain NCTC9081 (NCTC9081_02188), and *Treponema pallidum* strain TPANIC_082. Residues highlighted in red indicate amino acids involved in substrate binding as identified in *T. pallidum*. A residue highlighted in green indicates an essential substrate-binding site identified in *E. coli* and *N. meningitidis*. Residues involved in protein-substrate interaction, as identified in *N. meningitidis*, are marked in red. An asterisk indicates a residue conserved across all strains, a single dot marks residues shared by at least two strains, and two dots mark residues shared by three or more strains. Green arrows indicate the start and end points of the sequence of the recombinant MetQ, which is fused in its N-terminal extreme to MCGGHHHHHHHH, as encoded by the plasmid.
