# Supplementary material for: Identification of the methionine transporter MetQ in Streptococcus suis and its contribution to virulence and biofilm formation
Source: Vet Res. 2025 May 8;56:99. doi: 10.1186/s13567-025-01522-y (PMC12063423; doi:10.1186/s13567-025-01522-y)
Supplement: Supplementary file 3 — Additional file 3. Confirmation of the metQ knock-out mutant. (A) Schematic representation of the metQ gene context in S. suis strain P1/7 and P1/7ΔmetQ mutant, indicating the position of the primers used for PCR validation. Agarose gels showing the PCR products amplified using primers (B) metQ_L-Fw and metQ_R-Rev and (C) metQ_Fw and metQ_Rev primers in both the wildtype and ΔmetQ mutant. Primer sequences and predicted amplicon sizes are specified in Additional file 1. [file 13567_2025_1522_MOESM3_ESM.pdf]

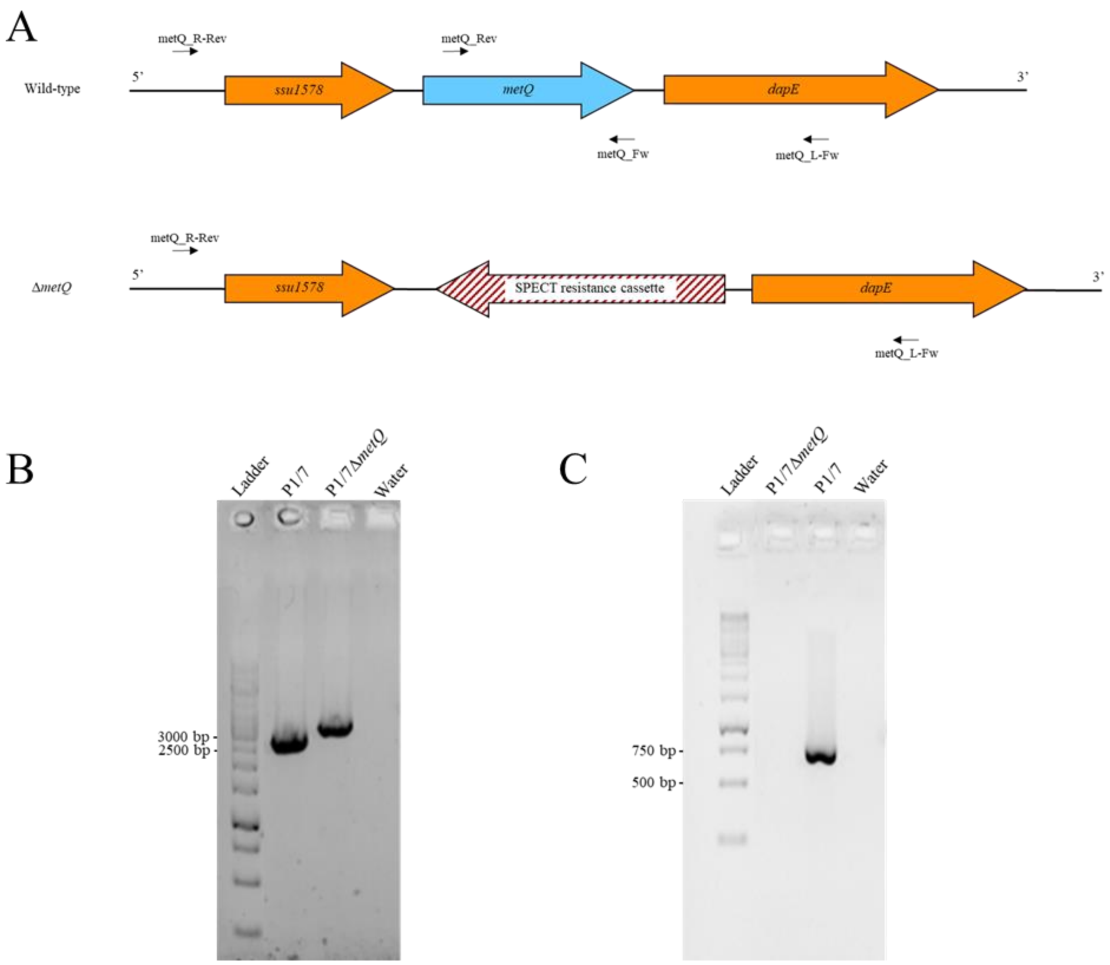

Additional File 3. Confirmation of the *metQ* knock-out mutant. (A) Schematic representation of the *metQ* gene context in *S. suis* strain P1/7 and P1/7Δ*metQ* mutant, indicating the position of the primers used for PCR validation. Agarose gels showing the PCR products amplified using primers (**B**) metQ_L-Fw and metQ_R-Rev and (**C**) metQ_Fw and metQ_Rev primers in both the wildtype and Δ*metQ* mutant. Primer sequences and predicted amplicon sizes are specified in **Additional File 1**.
