# Supplementary material for: Identification of the methionine transporter MetQ in Streptococcus suis and its contribution to virulence and biofilm formation
Source: Vet Res. 2025 May 8;56:99. doi: 10.1186/s13567-025-01522-y (PMC12063423; doi:10.1186/s13567-025-01522-y)
Supplement: Supplementary file 4 — Additional file 4. Production of recombinant MetQ. (A) SDS-PAGE assays showing whole cell lysates of BL21, BL21 carrying p16b-MetQ (BL21-pMetQ) grown in the presence or absence of IPTG and purified recombinant MetQ (rMetQ). (B) Western blot analysis of whole cell lysates from BL21, BL21-pMetQ grown in the presence or absence of IPTG and rMetQ using antiserum directed against a Histidine - tag. The position of rMetQ is indicated. Std (Standard Molecular weight). [file 13567_2025_1522_MOESM4_ESM.pdf]

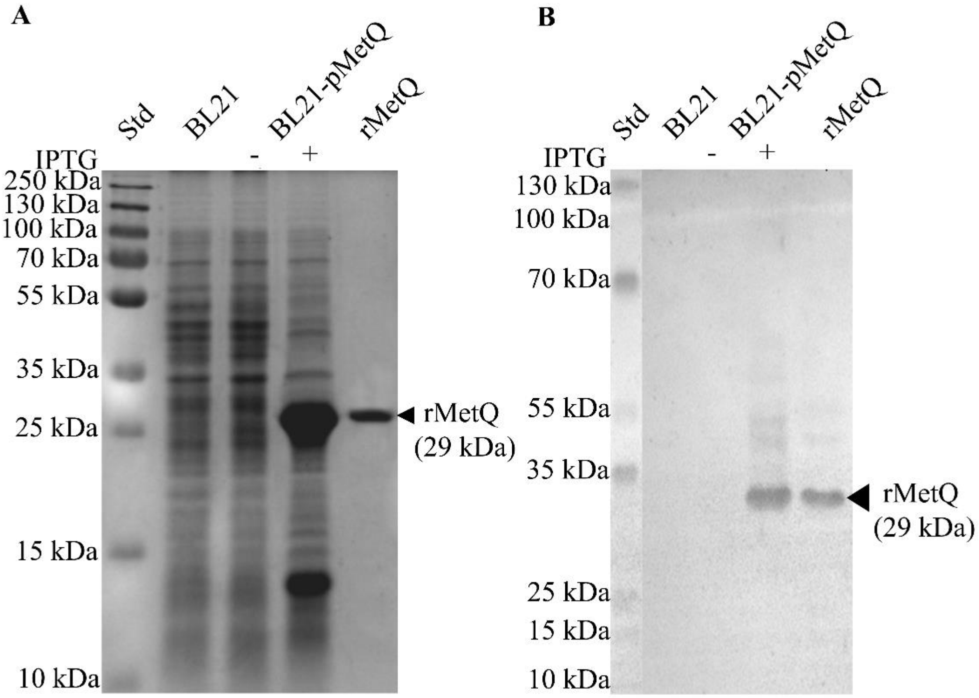

Additional File 4. Production of recombinant MetQ. (**A**) SDS-PAGE assays showing whole cell lysates of BL21, BL21 carrying p16b-MetQ (BL21-pMetQ) grown in the presence or absence of IPTG and purified recombinant MetQ (rMetQ). (**B**) Western blot analysis of whole cell lysates from BL21, BL21-pMetQ grown in the presence or absence of IPTG and rMetQ using antiserum directed against a Histidine - tag. The position of rMetQ is indicated. Std (Standard Molecular weight).
